# The size of via holes influence the amplitude and selectivity of neural signals in Micro-ECoG arrays

**DOI:** 10.1186/s42490-022-00060-4

**Published:** 2022-03-21

**Authors:** Manan Sethia, Mesut Sahin

**Affiliations:** Biomedical Engineering Department, New Jersey Institute of Technology, Newark, NJ 07102 USA

**Keywords:** Multi-electrode arrays, Perforation holes, Channel crosstalk

## Abstract

**Background:**

Electrocorticography (ECoG) arrays are commonly used to record the brain activity both in animal and human subjects. There is a lack of guidelines in the literature as to how the array geometry, particularly the via holes in the substrate, affects the recorded signals. A finite element (FE) model was developed to simulate the electric field generated by neurons located at different depths in the rat brain cortex and a micro ECoG array (μECoG) was placed on the pia surface for recording the neural signal. The array design chosen was a typical array of 8 × 8 circular (100 μm in diam.) contacts with 500 μm pitch. The size of the via holes between the recording contacts was varied to see the effect.

**Results:**

The results showed that recorded signal amplitudes were reduced if the substrate was smaller than about four times the depth of the neuron in the gray matter. The signal amplitude profiles had dips around the via holes and the amplitudes were also lower at the contact sites as compared to the design without the holes; an effect that increased with the hole size. Another noteworthy result is that the spatial selectivity of the multi-contact recordings could be improved or reduced by the selection of the via hole sizes, and the effect depended on the distance between the neuron pair targeted for selective recording and its depth.

**Conclusions:**

The results suggest that the via-hole size clearly affects the recorded neural signal amplitudes and it can be leveraged as a parameter to reduce the inter-channel correlation and thus maximize the information content of neural signals with μECoG arrays.

## Background

μECoG arrays can, for instance, monitor brain cortical activity in experimental animals, and localize the seizures in epilepsy patients, without penetrating the brain parenchyma. They are commercially available with metal contacts with varying sizes, inter-contact distances (pitch), and number of contacts on non-conductive substrate materials such as silicone, parylene-C, or polyimide [1]. Perforating via holes through the substrate are usually incorporated into the design to allow simultaneous recordings with penetrating microelectrodes [2] or injection of drugs [3]. However, the electrode array design is mostly based on personal experience of the investigator and putative design criteria that are believed to be the best match to the application in consideration. There is clearly a need for better guidelines as to how the array geometry affects the recorded signals. We hypothesized that the size of the via holes in particular must make a significant effect on the recorded signal amplitudes and perhaps on spatial selectivity since they provide passages of high electrical conductivity through a non-conductive substrate that forms an electrical barrier between the two sides of the array.

### Inter-Channel correlation

Several reports investigated the effects of contact spacing and size on the spatial selectivity of neural recordings. In a comparative study with μECoG recordings, the inter-channel correlation was found to be highly dependent on inter-contact distance and frequency of interest both in anesthetized human subjects and mice [4]. The smaller contact pitch with μECoG arrays (1.68 mm and 3 mm, in minipigs) was found to be instrumental for localization of the regions of evoked activity that were less than 1 cm apart, which would have not been possible with conventional ECoG electrodes [5]. On the other hand, a brain-computer interface (BCI) study in macaques concluded that the decoding performance decreased and inter-channel cross correlations increased when contact pitch was reduced from 9 mm to 3 mm [6]. Using FE models, the minimum contact pitch for high spatial selectivity was estimated to be 0.6 mm and 1.7 mm for the rat and human brains respectively for subdural placements of the arrays and using 10% of the max as a threshold to determine the spatial spread of the voltage [7].

### Signal amplitude and frequency content

Rigorous metrics were developed for comparing the quality of neural signals recorded with μECoG arrays in terms of their signal-to-noise ratio and frequency content [8]. A study conducted in resting human subjects showed that the μECoG arrays (75 μm contact diameter with 1 mm pitch) had significantly higher amplitudes and higher frequency components when the arrays were placed subdurally, compared to epidural placements, although the difference was negligible with macro ECoG arrays (2 mm contact diameter with 1 cm pitch) [9]. Subdural placement of μECoG arrays recorded frequency components up to 800 Hz on the rat cerebellar cortex where the inter-contact coherence increased substantially in transitioning from anesthesia to the awake state, whereas the frequency band from the motor cortex was limited to 200 Hz [10, 11], suggesting that the frequency content of the μECoG signals can vary substantially depend on the brain site and state. μECoG arrays with 100 μm contacts could detect multi-unit activity on the auditory cortex of guinea pigs [12]. It was also suggested that μECoG arrays cause less surgical complications during implantation than macro-ECoG due to their smaller size [13].

Although numerous reports looked at the effects of contact spacing and size on the recorded neural signal amplitudes, and spatial selectivity using both experimental data and FE analysis, as reviewed above, there is no significant study that systematically investigated the effects of perforating holes in the substrate that are commonly included in the μECoG designs, and the size of the substrate itself on the recorded signals. Computer simulations allow investigation of a large number of design variations in a reproducible manner free from uncontrollable perturbations and noise commonly seen in experimental setups, such as anatomical variations and differences in the implant quality between subjects. On the other hand, computer simulations may fall short of mimicking actual scenarios due to a lack of realistic values for specific conductivities of different tissue compartments, and due to local inhomogeneities that the computer models usually have to ignore to keep the computation time manageable. Nonetheless, the principles learned here and the underlying mechanisms should prevail across different μECoG designs although quantitative results may vary. In this paper, we used an FE model to gain some basic understanding of how the substrate size and the size of the via holes affect the amplitudes and spatial selectivity of the signals recorded from neurons located in the gray matter of a rat brain.

## Methods

**Fig. 1:**
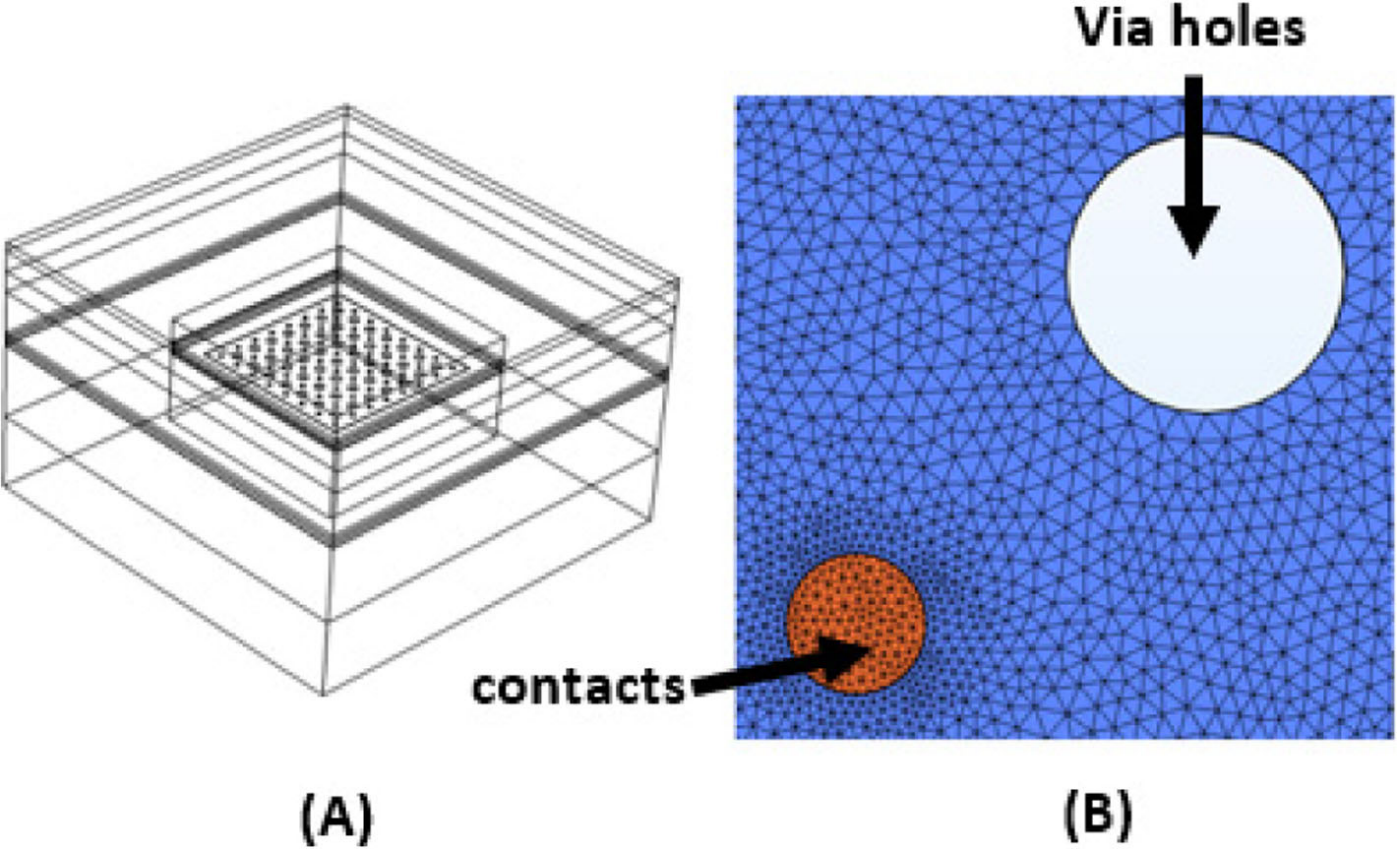
Finite element model of a rat brain cortex containing a μECoG electrode array positioned on the pia mater. **A**: A small box (5x5x2.5 mm) with extremely fine mesh is defined inside a larger one (10x10x6.1 mm). **B**: Detailed mesh view around a metal contact (100 μm, orange circle) and a via hole (200 μm, white circle) is shown

**Fig. 2:**
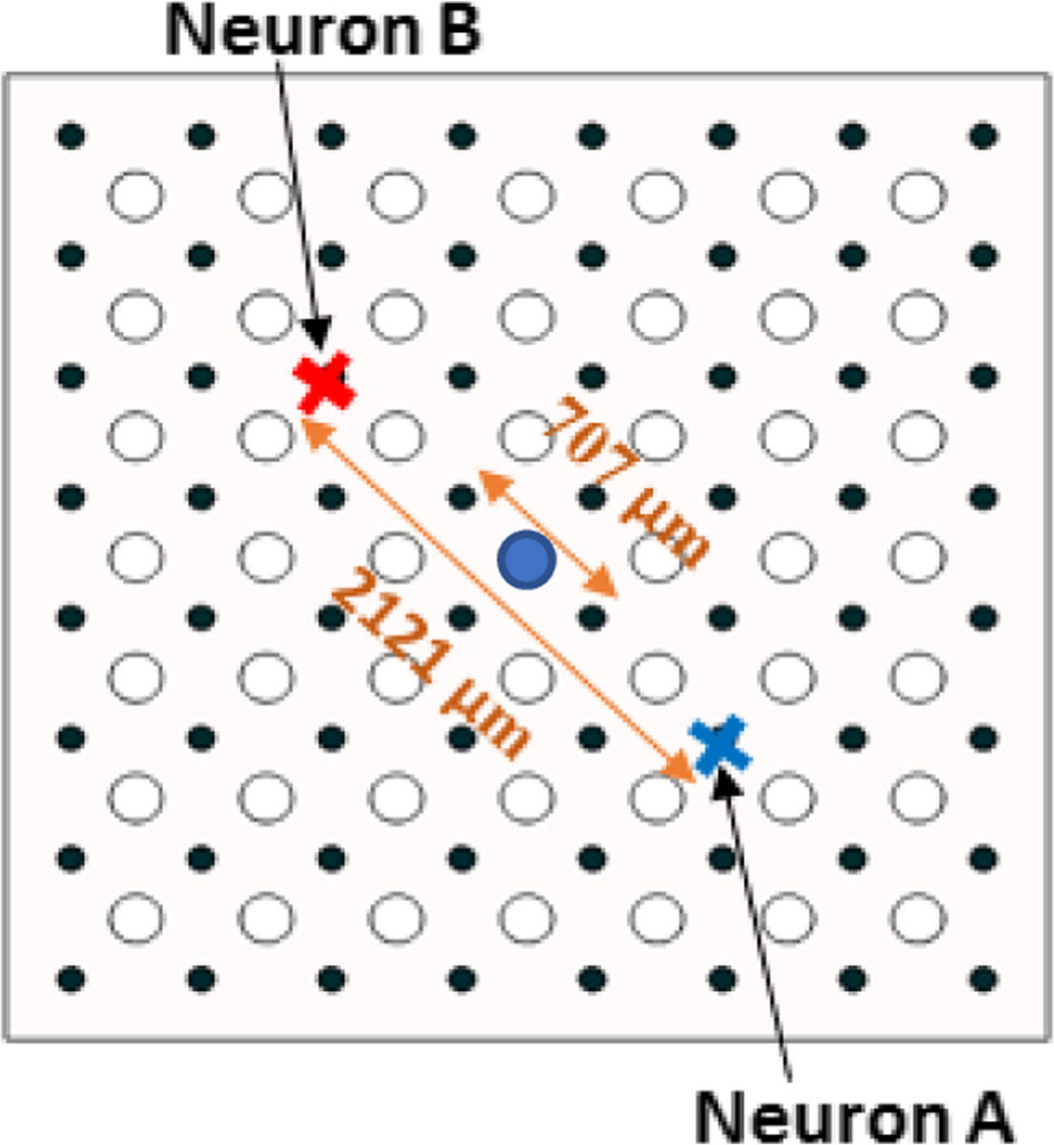
Arrangement of the 8 × 8 contacts (filled circles) and the via holes (open circles) in the array. The center-to-center distance between the via holes and the contacts is 500 μm in the horizontal and vertical directions. For selectivity analysis, the off-center neurons, Neuron A and B, are either aligned with the contacts that are 2121 μm apart as shown with crosses, or with the contacts on each side of the central hole that are 707 μm apart. The via hole in blue is at the array center

A FE model was developed on COMSOL Multiphysics v5.4 platform. The model was designed to mimic a rat brain in terms of layer thicknesses and their electrical conductivities taken from the literature (\ 1). The model was divided into ten isotropic layers representing air, scalp, skin, skull, dura mater, arachnoid, sub-arachnoid or cerebrospinal fluid (CSF), pia mater, gray matter and the white matter (Table 1). The μECoG electrode design was constructed with typical parameters (Table 2) commonly used in commercially available 8 × 8 arrays for animal experiments [14, 15]. The contacts were 100 μm in diameter with a pitch of 500 μm (Figs. 1 & 2) placed on a polyimide substrate with a thickness of 20 μm. As it is commonly included in the μECoG array designs, via holes with varying sizes (20 μm, 50 μm, and 200 μm) were introduced into the substrate at the geometric center of each set of four neighboring contacts (Fig. 2). A neuron was modelled using a dipole current source with a magnitude of 1 μA and a separation of 50 μm, and vertically positioned at one of the three different depths; 500 μm, 1000 μm, and 1500 μm, from the pia surface. Note that 1 μA was adopted here as a generic value, and not intended to mimic the membrane current of a specific type of a neuron. Thus, the voltages reported here should be considered as relative numbers with respect to 1 μA source current. Voltage profiles were simulated also for the cases of no via holes and in the absence of an electrode substrate for comparison.

**Table 1:** Thicknesses and electrical conductivities for the cortical layers included in the rat brain model

Rat Brain Model			
Layer	Thickness (μm)	Specific conductivity (S/m)	References
Air	100	1 × 10^− 15^	
Scalp	500	0.2	Geddes and Baker 1967
Skin	500	0.05	
Skull	1000	0.02	Kosterich et al.
Dura	100	0.03	Struijk et al., 1997
Arachnoid	75	0.03	
CSF	100	1.8	Baumann et al., 1997
Pia	25	0.23	
Gray	1800	0.23	Latikka et al., 2001
White	1800	0.6	Ranck and Bement, 1965

**Table 2:** Material properties and geometric parameters of the μECoG array

μECoG array design		
Contacts platinum/ iridium (4x10^6^S/m)	contact diam.	100 μm
	contact pitch	500 μm
	No. of contacts	8 × 8 contacts
Substrate polyimide (6.67 × 10^− 16^ S/m)	substrate dimensions	0.5 × 0.5 mm 1 × 1 mm 2 × 2 mm 4 × 4 mm
	thickness	20 μm

Boundary conditions were applied to the model by assigning ground terminal to all the outer boundaries except the top surface, which was by default assigned as an insulator (air). A smaller cubical box (5x5x2.5 mm) was constructed containing the μECoG array and the neuron, and was set to “extremely fine” level of mesh (element size 2 μm). The middle four layers from the pia to dura were set to “extra fine” mesh (element size 15 μm) and the gray matter outside the small box, the white matter, and the top four layers were set to “finer” mesh (element size 40 μm). The model consisted of ~ 11.6 million domain and boundary elements, and the simulation time was ~ 1 h 20 min on a i5-8265U dual-core CPU running at 1.60GHz and 1.80 GHz with 8 GB RAM. Voltages computed at all the elements of the 3D COMSOL model were exported to Matlab (Mathworks Inc.) and voltage profiles next to the bottom surface of the substrate were plotted. The presence of the metal contacts made small differences in the voltage profiles. The voltage at any point underneath the array could also be thought of as a voltage measurement point from an infinitely small contact hypothetically located at that point. We noticed that electric fields at the vicinity of the contacts had significant computational errors due to sharp transitions in conductivity in a span of a few microns. Thus, for accurate calculations of spatial selectivity, the contacts were removed from the model and the voltages were taken where the contacts were located in the original model. In real electrodes, the presence of the contacts will not affect the voltage field to the degree predicted by the FE model because of the electrode-electrode interface impedance distributed across the contact surface.

When contacts were present, the voltage profiles made across the array were sampled at 10 μm below the array surface to avoid these transitional effects.

A neuron was placed beneath the central via hole at varying depths for initial amplitude analysis (Fig. 2). For selectivity analysis, two neurons symmetrically positioned along the array’s diagonal axis and with varying depths were introduced to the model. Spatial selectivity is the ability of an electrode to record preferentially higher signals from one neuron vs. another at a different location. For example, Neuron A positioned precisely below the recording contact will induce a higher amplitude signal on this contact compared to another neuron (Neuron B) placed farther away (Fig. 2). Thus, spatial selectivity (SS) is defined as the ratio of the potential difference between the voltages induced by those two neurons to the voltage of the neuron that is located closer to the recording site. A SS value of 1 represent perfect selectivity where V_B_, signal from Neuron B, is zero.

1 $$ SS=\frac{V_A-{V}_B}{V_A} $$

## Results

**Fig. 3:**
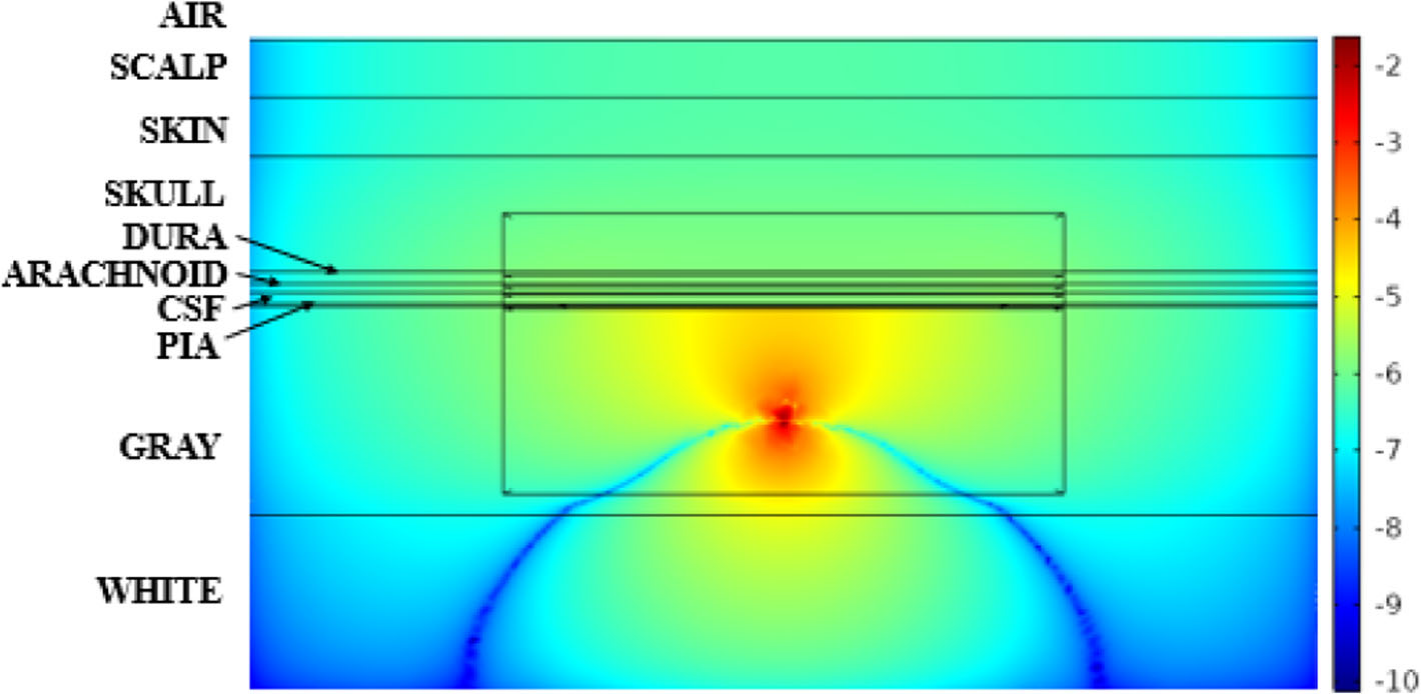
Voltage field in a vertical plane that cuts through the center of the model. Absolute values of the voltages are plotted on a logarithmic scale shown on the right. The neuron is located where the maximum voltages are observed. The small box delineates the region with extremely fine mesh containing the array and the neuron. Both positive (above the neuron) and negative (below the neuron) voltages are shown on the same color scale using the absolute values

As expected, the voltage field of the neuron, simulated as a dipole current source, drops exponentially by distance (Fig. 3). Near zero potentials are measured (dark blue areas) in regions where the anodic and cathodic fields from the dipole cancel each other.

The voltage field spreads further above the neuron than it does below it due to the presence of a low-conductivity skull and the non-conductive air above the scalp. The electrode array also blocks the vertical flow of the current, which further reduces the voltage gradient (rate of decrease) in the vertical direction.

**Fig. 4:**
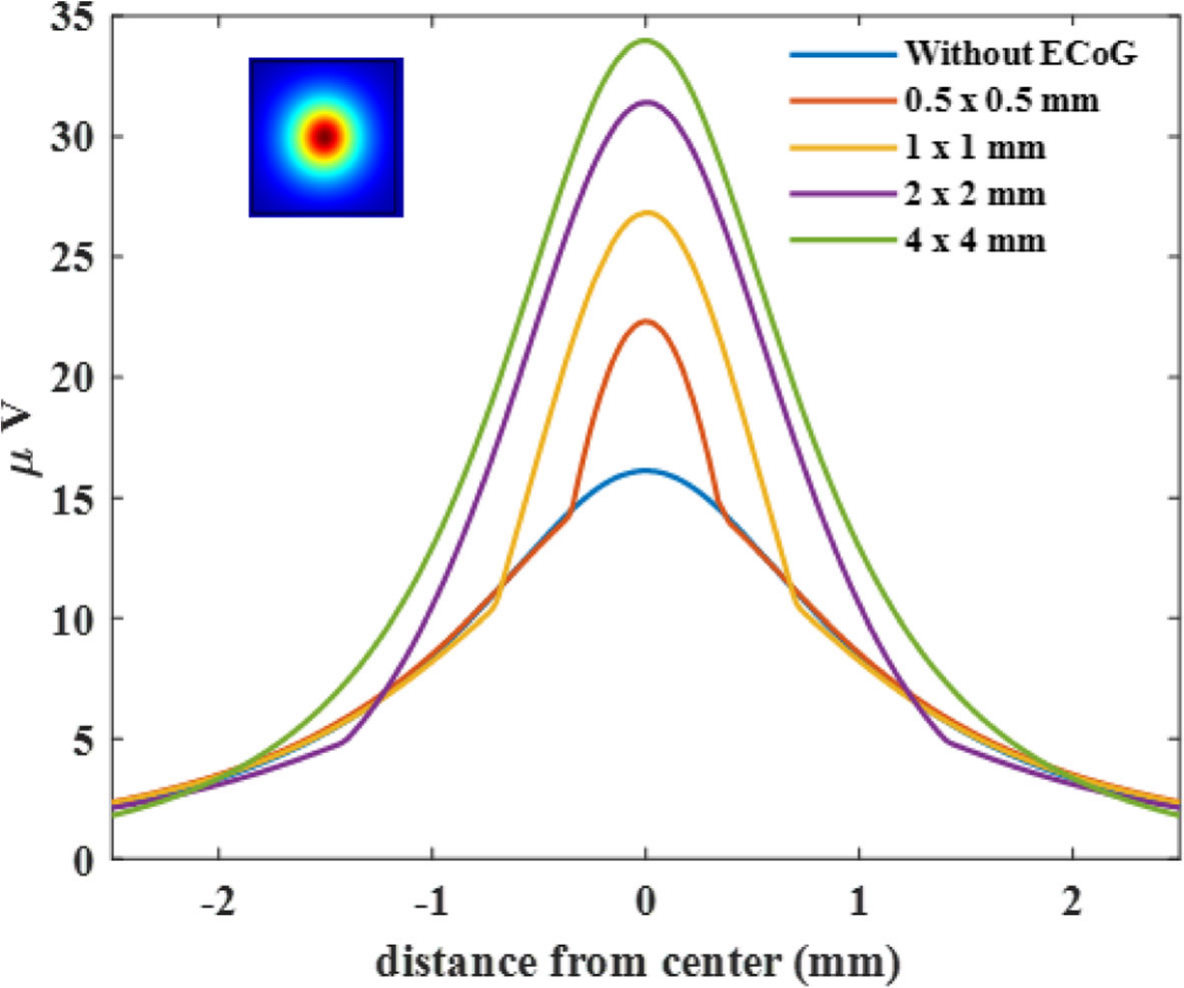
Voltage profile along the diagonal axis beneath the electrode array for varying substrate sizes as well as in the absence of a substrate. The recorded voltage increases with array size. The neuron is at a depth of 1000 μm from the pia surface and aligned with the center of the array. No via holes

In order to demonstrate the effect that the presence of a non-conductive substrate makes on the recorded voltages, the voltage profiles at the substrate bottom surface were plotted for different substrate sizes (Fig. 4). The voltage amplitudes increased under the array and decreased outside the substrate compared to the no-substrate case (blue trace). Consequently, the voltage profiles had sharp slope changes at the edges of the substrate. The peak voltage increased significantly with the substrate size and reached to ~ 34 μV for the 4 × 4 mm array (green trace), which was larger than twice the voltage recorded in the absence of the array (16 μV, blue trace). The peak voltage for a 10 × 10 mm substrate (not shown) was close to that of 4 × 4 mm, indicating a plateau effect. For the substrate size of 1 × 1 mm, which is in the same order as the neuron depth in this case (1000 μm), the voltage (26.8 μV) increase was about 68% compared to the no-substrate case, and 21% less than the voltage measured with the largest substrate (34 μV). These simulations suggest that the presence of a non-conductive array substantially impacts the voltages recorded at the array, especially when the substrate size is a few times larger than the depth of the neuron that is acting as the source.

**Fig. 5:**
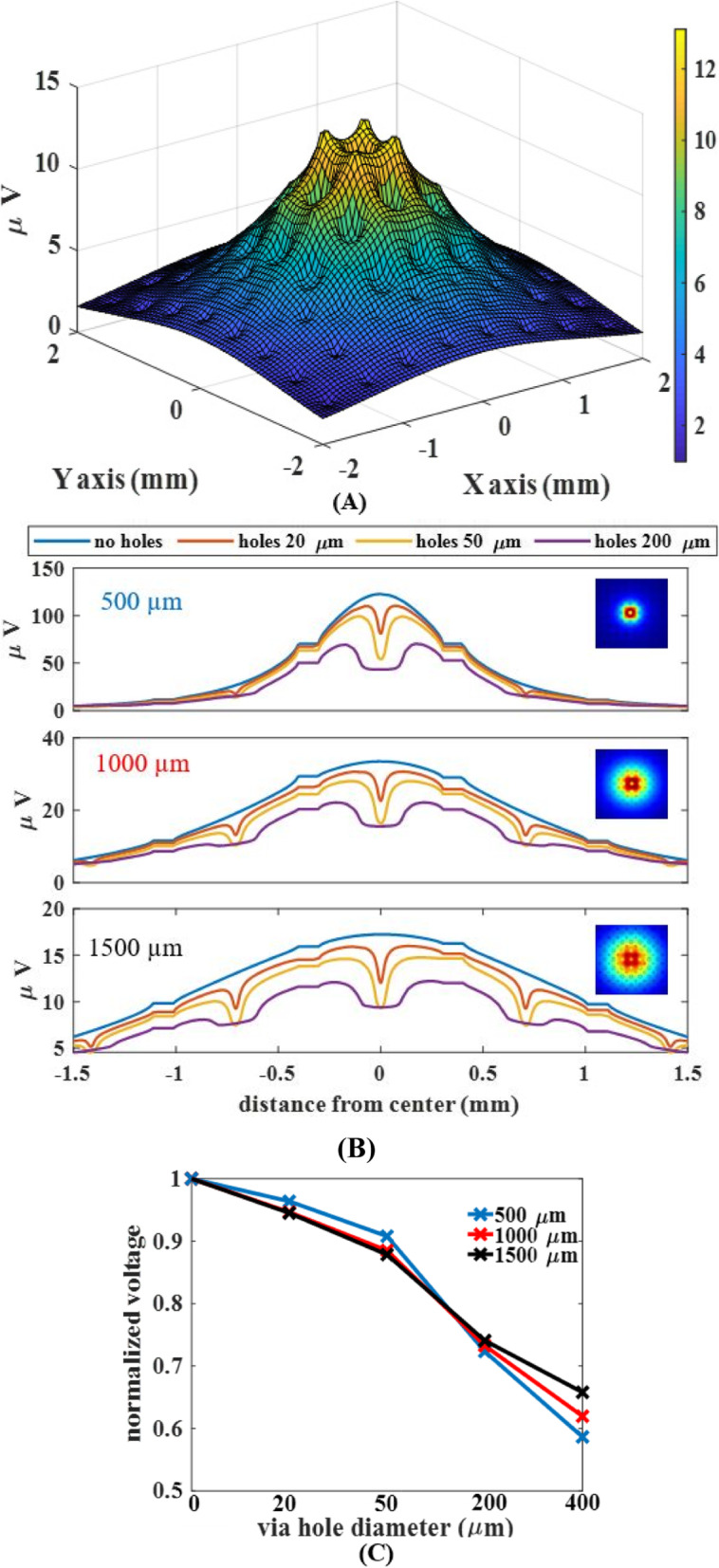
Voltage profiles recorded at the bottom surface of the array (4 × 4 mm) from neurons located at three different depths (500 μm, 1000 μm, and 1500 μm) for different via hole sizes. **A**: Surface mesh plot depicting the effects of the via holes (200 μm) on the voltage profile from a neuron located at a depth of 1500 μm exactly beneath the geometric center of the array. **B**: Voltage profiles along the diagonal axis of the array that goes through the centers of the holes and contacts. **C**: Relative amplitudes (recorded at the contact locations that are closest to the via hole at the array center) for different via hole sizes. Each trace is normalized by the voltage for the substrate with no holes

The effect of the hole size was investigated for different neuron depths (Fig. 5). The 3D mesh plots in Fig. 5A resemble a spongy bed-like structure with a peak voltage at the horizontal coordinates of the source neuron. The voltage amplitudes decreased and spread wider for the neurons positioned deeper into the gray matter from the pia surface (Fig. 5B). The fractional voltage drops at the center of the holes were similar for all neuron depths, although the absolute values were smaller for deeper neurons. The electrode contacts introduced horizontal steps in the profile by forcing the local potential to the average of the voltages around them because of their high conductivity. Interestingly, the voltages at locations that are away from the via holes were also affected and deviated from the voltage profiles of the “no holes” case to increasing degrees with the hole size.

Normalized voltage profiles demonstrated an interesting interplay between the neuron depth and the hole size on the recorded amplitudes (Fig. 5C). The relative impact of the via holes was smaller first for the neuron closest to the surface (500 μm) and then larger than that of deeper neurons as the hole size was increasing. Thus, the curve is steeper for shallower neurons.

**Fig. 6:**
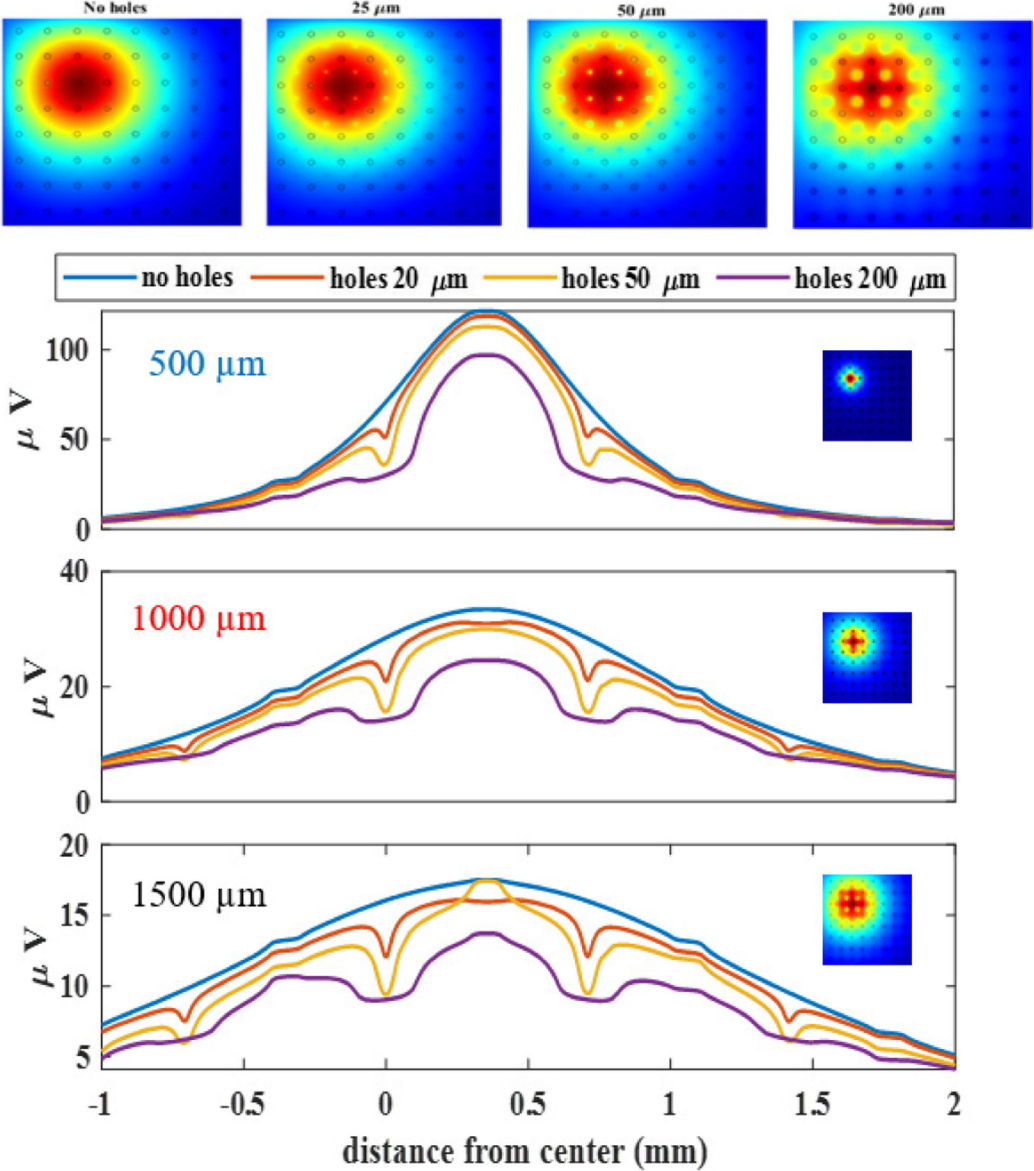
Voltage profiles for off-center positioning of the neuron. Top panel: 2D versions of the voltage fields for different sizes of the via holes; Bottom Panel: Voltage profiles recorded from neurons at three different depths (500 μm, 1000 μm, and 1500 μm) and via-hole sizes, at the bottom surface of the electrode array (4 × 4 mm) along the diagonal axis that goes through the centers of the holes and contacts (see Fig. 2). The heat maps for the corresponding neuron depths are shown as insets on the right. 0 mm is the array center

Next, the neuron was moved off-center and aligned with a contact on the diagonal axis (Neuron A in Fig. 2) in order to visualize the effects of asymmetry on the recorded signals (Fig. 6). The asymmetry induced in the voltage distribution due to the array edges closer to the neuron can be appreciated in the heat-plots of the top panel. The plots in the bottom panel resemble those in Fig. 5B except that there is a contact at the location of the voltage peak instead of a via hole. Unlike the plots of Fig. 5B, however, the positioning of the neuron produced a slight asymmetric in the voltage profiles, which was more pronounced with the neurons closer to the surface.

**Fig. 7:**
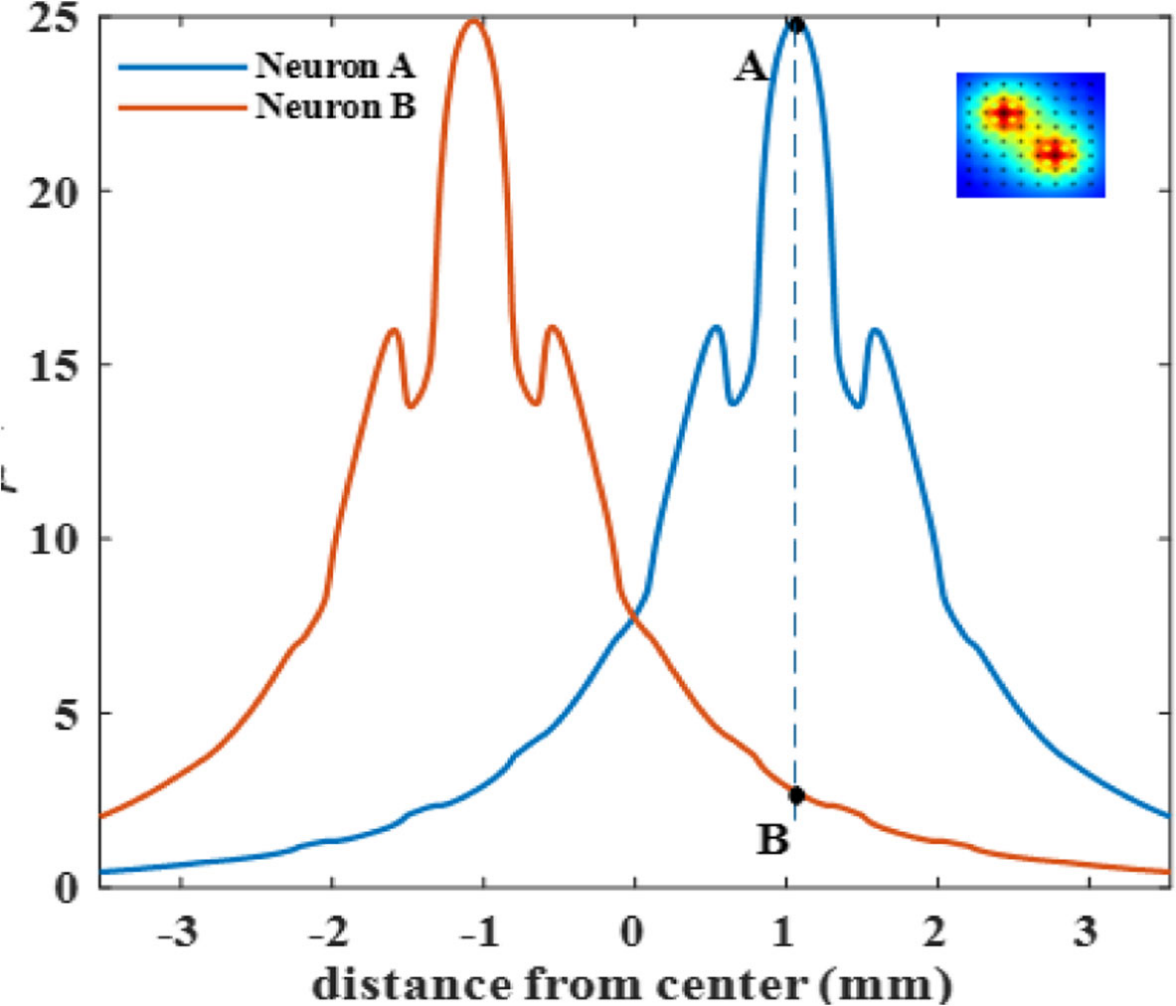
Voltage profiles recorded by the contact above Neuron A from both Neuron A (blue trace) and Neuron B (red trace) that are located across the array’s diagonal line 2121 μm apart (as shown in inset and Fig. 2). Depths of neurons = 1000 μm, hole sizes = 200 μm. The dash line marks the location of the recording contact that is directly above Neurons A

Next, we investigated spatial selectivity. Figure 7 illustrates the voltages recorded by the contact positioned directly above Neuron A from that neuron and also Neuron B, which is symmetrically positioned on the other side of the array center as shown in Fig. 2. Spatial Selectivity is defined by Equ. 1 where A and B are the voltages recorded from Neuron A and Neuron B respectively as marked by black dots in Fig. 7. Voltage profiles are slightly asymmetrical as expected. In this example for a specific neuron depth and hole size, the selectivity is 0.88.

**Fig. 8:**
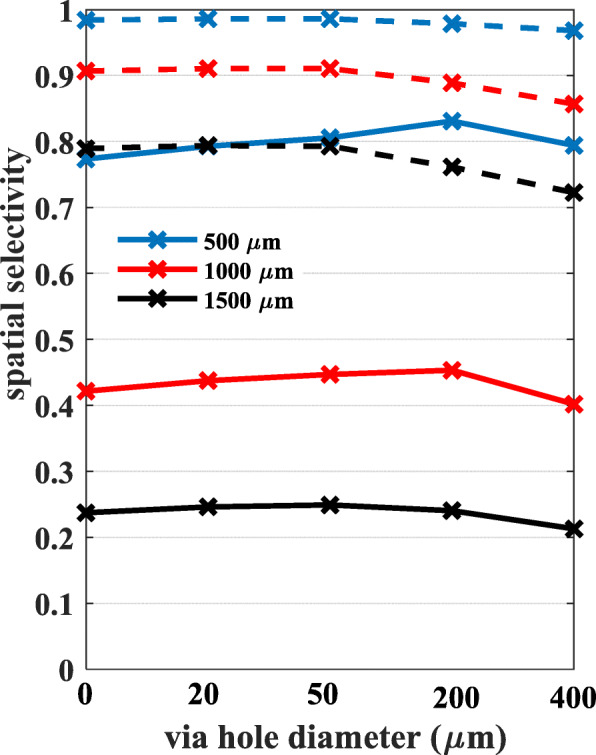
Spatial selectivity values calculated for neuron pairs located at different depths (500, 1000, and 1500 μm) and for different sizes of the via holes. The distance between Neuron A and B is either 707 μm (solid lines) or 2121 μm (dash lines) as shown in Fig. 2. In all cases, the signals were recorded at the contact that is aligned with Neuron A

Spatial selectivity is lower for neurons located deeper in the gray matter (Fig. 8). The presence of the holes lowers the selectivity with a stronger impact as the hole size is increasing when the distance between the neurons is large (2121 μm, dash lines) regardless of the neuron depth. Paradoxically, the selectivity increases with increasing hole sizes initially when the inter-neuron distance is smaller (707 μm) before it drops for the larger hole size(s). Spatial selectivity is maximized at 200 μm via-hole size for neuronal depths of 500 μm and 1000 μm, and at 50 μm hole size for the deepest neuron (1500 μm).

## Discussion

### Substrate size

The presence of the substrate blocks the currents flowing in the vertical direction and thus reduces the rate of voltage decline by distance from the neuron in that direction. The size of the electrode substrate clearly improves the recorded signal amplitudes especially if it is larger than the array-neuron distance. The signal amplitude saturates once the array dimensions are an order of magnitude larger than the depth of the neuron. The thickness of the human cortex varies between 1 and 4.5 mm and has an average thickness of 2.7 mm on the gyral regions [16].

The human versions of the ECoG arrays are usually at least an order of magnitude larger than the deepest targets in the cortex. Thus, the clinical arrays that are larger than a few cm square should be able to maximize the signals even from the deepest neurons in the cortex due this substrate effect. However, as the brain size is becoming smaller in smaller species like the rat and mouse, the cortex thickness does not scale down proportionally, and sometimes small arrays are preferred with dense arrangement of the contacts. As a practical value, one should be aware that the signal amplitudes may be reduced down to 79% (of the amplitudes recorded with a large array) when the substrate dimensions are in the same order as the depth of the targeted neurons (compare 1 × 1 mm and 4 × 4 mm arrays in Fig. 4).

### Holes size as a design parameter

Intuitively, the effect of the via holes should be the opposite to that of the substrate. We can anticipate that the presence of a large via hole in the substrate should cause some reduction of the recorded signals. That is, the current flowing through the holes would increase the rate of decline in the extracellular voltage in the vertical direction. The simulations of the current study provided some general guidelines on how the signal amplitudes vary with the hole size. The impact slightly depends on the depth of the targeted neurons for recording. As a practical guideline, 200 μm holes will cause about 25% reduction in the recorded signals from the rat cortex, regardless of the neuron depth, compared to the case without via holes (Fig. 5C). This hole size is about 40% of the contact pitch (500 μm). But, the signal deterioration will be stronger for shallower neurons if the via-hole size is larger than 200 μm.

### Spatial selectivity

If the inter-neuron distance is large (dash lines in Fig. 8), spatial selectivity is large to begin with but lowered by increasing amounts with the hole size and the neuron depth. For shallow neurons, the effect is negligibly small even at the largest hole size of 400 μm (80% hole diam. / pitch ratio). For smaller inter-neuron distances (solid lines in Fig. 8), selectivity first increases with the hole size before the point of diminishing returns, which occurs at smaller via-hole sizes for deeper neurons. In general, it seems that inter-neuron distance and neuron depth have opposing effects on selectivity, and the hole size is a third parameter that can maximize selectivity at a point determined by the first two. Overall, potential improvement on selectivity by optimizing the hole size is marginal. Larger improvements in selectivity may be possible with alternative arrangements of the contacts and the holes on the substrate. Nonetheless, even this marginal gain in selectivity may provide the edge needed when multi-contact arrays are used for source localization in different layers of the brain cortex. Finally, we have to point out that micro vessels and connective tissue may grow through the perforating holes over time in chronic implants [17]. This may significantly reduce the field effects induced by the presence of the holes due to somewhat higher resistivity of these tissues than the CSF, which would otherwise be filling the holes.

## Conclusions

Spatial selectivity of multi-contact neural recordings could be maximized by proper selection of the via-hole size. Increasing the spatial selectivity is analogous to reducing the inter-channel correlation and thus maximizing the information content of the multi-channel signals. The via-hole size could be leveraged as an optimization parameter to maximize the information content of neural recordings while maintaining sufficient signal amplitudes above the noise floor. Further investigation of this phenomenon is warranted within a larger parameter space and using more realistic neural models that include all neuronal compartments such as a dendritic three, the soma, and an axon with realistic membrane currents and positions in the gray matter. The optimum via-hole size may also be different for different neuronal subtypes because of differences in their morphology and orientation, in addition to depth, in the gray matter.

## Data Availability

No biological data included in this publication. The finite element model can be made available upon request vial email at sahin@njit.edu.

## Abbreviations

ECoG: Electrocorticography
μECoG: Micro-electrocorticography
FE: Finite element
BCI: Brain-computer interface
CSF: Cerebrospinal fluid
